# Prevalence, diagnostic delay and economic burden of endometriosis and its impact on quality of life: results from an Eastern Mediterranean population

**DOI:** 10.1093/eurpub/ckad216

**Published:** 2023-12-09

**Authors:** Bethan Swift, Bahar Taneri, Christian M Becker, Hasan Basarir, Huseyin Naci, Stacey A Missmer, Krina T Zondervan, Nilufer Rahmioglu

**Affiliations:** Oxford Endometriosis CaRe Centre, Nuffield Department of Women’s and Reproductive Health, University of Oxford, Oxford, UK; Wellcome Centre for Human Genetics, University of Oxford, Oxford, UK; Faculty of Arts and Sciences, Department of Biological Sciences, Eastern Mediterranean University, Famagusta, Northern Cyprus; Cyprus Women’s Health Research Society (CoHERS), Nicosia, Northern Cyprus; Oxford Endometriosis CaRe Centre, Nuffield Department of Women’s and Reproductive Health, University of Oxford, Oxford, UK; RTI Health Solutions, Manchester, UK; Department of Health Policy, London School of Economics and Political Science, London, UK; Department of Obstetrics and Gynecology and Reproductive Biology, College of Human Medicine, Michigan State University, Grand Rapids, MI, USA; Department of Epidemiology, Harvard T.H. Chan School of Public Health, Boston, MA, USA; Oxford Endometriosis CaRe Centre, Nuffield Department of Women’s and Reproductive Health, University of Oxford, Oxford, UK; Wellcome Centre for Human Genetics, University of Oxford, Oxford, UK; Oxford Endometriosis CaRe Centre, Nuffield Department of Women’s and Reproductive Health, University of Oxford, Oxford, UK; Wellcome Centre for Human Genetics, University of Oxford, Oxford, UK; Cyprus Women’s Health Research Society (CoHERS), Nicosia, Northern Cyprus

## Abstract

**Background:**

There are limited data on endometriosis from the Eastern Mediterranean region. This study for the first time estimates the prevalence and impact of endometriosis on women in Northern Cyprus, an under-represented region in Europe.

**Methods:**

Cyprus Women’s Health Research Initiative, a cross-sectional study recruited 7646 women aged 18–55 in Northern Cyprus between January 2018 and February 2020. Cases were identified using self-reported and ultrasound data and two control groups were defined, with (*n* = 2922) and without (*n* = 4314) pain. Standardized tools, including the 11-point Numerical Rating Scale and the Short Form 36 Health Survey version 2, were used to assess pain and quality of life, respectively.

**Results:**

Prevalence and median diagnostic delay of endometriosis were 5.4% [95% confidence interval (CI): 4.9–5.9%, *n* = 410] and 7 (interquartile range 15.5) years. Endometriosis cases experienced a higher prevalence of bladder pain compared with asymptomatic pain controls (6.3% vs. 1.0%, *P* < 0.001) and irritable bowel syndrome relating to pelvic pain compared with symptomatic (4.6% vs. 2.6%, *P* = 0.027) and asymptomatic (0.3%, *P* < 0.001) controls. The odds of endometriosis cases reporting an anxiety diagnosis was 1.56 (95% CI: 1.03–2.38) higher than the symptomatic and 1.95 (95% CI: 1.30–2.92) times higher than the asymptomatic controls. The physical component score of the health-related quality-of-life instrument suggested a significant difference between the endometriosis cases and the symptomatic controls (46.8 vs. 48.5, *P* = 0.034). Average annual economic cost of endometriosis cases was Int$9864 (95% CI: $8811–$10 917) including healthcare, costs relating to absence and loss of productivity at work.

**Conclusion:**

Prevalence was lower than the global 10% estimate, and substantial proportion of women without endometriosis reported moderate/severe pelvic pain hinting at many undiagnosed cases within this population. Coupled with lower quality of life, significant economic burden and underutilized pain management options, the study highlights multiple opportunities to improve care for endometriosis patients and women with pelvic pain.

## Introduction

Endometriosis is a chronic gynaecological condition characterized by the presence of endometrial-like tissue in locations other than uterus, such as ovaries, bladder and bowel.^1^ The true prevalence of the disease is unknown as estimates are affected by the need for mainly surgical diagnostic methods^2^ and on characteristics of the study population but is estimated to be around 5–10%.^3^ In addition, observed differences in prevalence rates across populations may be caused by differential access to healthcare.^3^ Endometriosis is difficult to diagnose because for definitive diagnosis most of the time direct visualization is required during laparoscopy, which is a costly and invasive procedure.^4^ Significant advancements in quality and availability of non-invasive imaging techniques mean that laparoscopy is no longer recommended as the sole gold standard.^5^ However, imaging techniques have limited ability to detect superficial endometriosis so cannot replace laparoscopy for detection of all endometriosis subtypes.

Symptom heterogeneity of endometriosis is high; the most common symptoms are dysmenorrhoea, dyspareunia, non-cyclic pelvic pain and infertility, although a large proportion of women are asymptomatic. An additional difficulty with diagnosing endometriosis is that symptoms can also be attributed to other conditions such as those relating to the bladder or bowel.^6^ These difficulties with diagnosis mean that on average, women consult a total of seven clinicians before receiving a diagnosis of endometriosis.^7^ The Global Study of Women’s Health (GSWH)^8^ included women from 10 countries who were undergoing their first laparoscopy for symptoms suggestive of endometriosis and showed that average diagnostic delay ranged from 3 to 10 years.

The chronic, complex and debilitating nature of endometriosis means women with the condition report lower health-related quality-of-life (HRQoL) scores, both on physical and mental domains.^9^ This finding was also observed in the GSWH, which suggested women who experienced longer diagnostic delays reported a lower HRQoL score.^8^ The healthcare costs have been estimated to be similar to other chronic diseases such as diabetes and heart disease^10^ and the GSWH estimated the average yearly cost of endometriosis to be €9579, while a study in Australia found that the average cost was Int $20 898 with costs of productivity loss being the biggest contributor.

Although multi-centre studies have aided the understanding of the estimated prevalence and quality of life associated with endometriosis, there is limited such research from Eastern Mediterranean populations. Cyprus is the third largest Mediterranean island home to approximately 300 000 Turkish Cypriot and 700 000 Greek Cypriot residents. Due to political circumstances in the region, there is a lack of population health data from the Turkish Cypriot population in Northern Cyprus.^11^

The objectives of this study were to estimate the prevalence, diagnostic delay and economic burden among women in Northern Cyprus and investigate the impact of disease and its associated symptoms on HRQoL.

## Methods

### Participants

This study used data collected as part of the Cyprus Women’s Health Research (COHERE) Initiative,^12^ a population-based cross-sectional study that recruited 7646 women aged 18–55 in Northern Cyprus between January 2018 and February 2020. Our sample was broadly representative of the population,^13^ compared with the 2019 census estimates for Northern Cyprus.

Participants completed the World Endometriosis Research Foundation EPHect (WERF EPHect) questionnaire.^14^^,^^15^ In addition, the questionnaire included the Short Form 36 Health Survey version 2 (SF-36v2),^16^ the Work Productivity and Activity Impairment: General Health (WPAI: GH),^17^ the University of California San Diego (UCSD) migraine questionnaire,^18^ the Pain Catastrophizing Scale (PCtS),^19^ the Short form McGill Pain Questionnaire (SF-MPQ)^20^ and the Rome IV diagnostic criteria for IBS.^21^

As part of this study, all women were invited to sign up for an optional clinical visit which included a transvaginal/transabdominal pelvic ultrasound scan (USS).

### Endometriosis prevalence and case ascertainment

Endometriosis cases were those who self-reported to have endometriosis when asked ‘has a doctor or other healthcare provider ever diagnosed you with endometriosis’ and those women found to have evidence of an endometrioma at their clinical visit during the USS.

Women were defined as suffering from severe pain if they scored higher than 4 on the 11-point numerical rating (0–10) scale (NRS)^22^ for at least one of the following questions: dysmenorrhoea—‘severity of period pain at its worst in the last 12 months’, dyspareunia—‘severity of pain at its worst during the last time you had vaginal intercourse/penetration or in the 24 hours after the last time you had vaginal intercourse/penetration’ and a cyclic pelvic pain—‘severity of pelvic pain at its worst in the last 3 months’.

Two control groups were created to explore whether certain symptoms or characteristics were due to endometriosis *per se*, or due to the pain that endometriosis cases were experiencing: (i) symptomatic pain control; women who did not report to have endometriosis but reported to have dysmenorrhoea, dyspareunia or non-cyclic pelvic pain and reported the severity to be >4 on the relevant NRS as described above and (ii) the remainder of the cohort, i.e. those who did not report the severity of dysmenorrhoea, dyspareunia or pelvic pain to be >4.

### Diagnostic delay

Diagnostic delay was calculated by subtracting the age of symptom onset from the age of diagnosis. Hypothesizing that symptom awareness of endometriosis was low in this population, an alternate age of symptom onset variable was created by examining the earliest age women self-reported to have either dysmenorrhoea, non-cyclical pelvic pain or dyspareunia. These pelvic pain types are the most common symptoms of endometriosis.^1^ An alternative diagnostic delay variable was created by subtracting the alternate age of symptom onset variable from the age of endometriosis diagnosis.

### Associated pain conditions

The SF-MPQ was utilized to explore subcategorization of pain symptoms based on two separate domains: sensory and affective; the PCtS was used to investigate the rumination, magnification and helplessness of the presence of pain. The Rome IV criteria allowed assessment of those suffering from IBS symptoms during the menstrual cycle and during an episode of pelvic pain. The UCSD was used to assess the prevalence of migraine. Bladder pain was defined as those women who rated their bladder pain at its worst over the last 7 days as above 4 on the NRS.

### Quality of life in endometriosis

We utilized the SF-36v2 questionnaire, validated in Turkish,^23^ to investigate the relationship between endometriosis and HRQoL.^24^ The eight physical and mental subscale scores, as well as the two overall summary measures for physical and mental health, known as the physical component summary (PCS) and mental component summary scores were calculated.^16^^,^^25^ We investigated differences in quality of life by stratifying for experience of pain.

### Psychological disorders

Self-reported diagnosis of anxiety and depression was extracted from the questionnaire with non-responses coded as not received a diagnosis. Prevalence of anxiety and depression was calculated as number of self-reports over the total number of women in each case or control group.

### Work productivity loss and economic burden

Analysis of the WPAI: GH followed standard methods for the calculations of the dimensions.^17^ To calculate the cost of endometriosis and its associated symptoms, we included direct healthcare costs^26^ and indirect costs related to time off work or loss of productivity while at work. Since this was a prevalence analysis, costs were estimated regardless of the time of diagnosis. Costs were estimated in Turkish Lira using 2022 prices and then converted into International Dollars (Int$), which are tied to the US dollar, by dividing the price in Turkish Lira by 2.61, which was the conversion factor taken from the World Bank at the time of analysis.^27^ Costs were extrapolated to estimated costs over one year. For detailed methodology, please see Supplementary information.

### Statistical methods

Statistical analyses were carried out using Stata SE v17.0 (StataCorp LP, USA). Descriptive variables were summarized using frequencies and differences between groups tested using Chi-square and Fisher’s exact test for categorical variables, and independent sample *t*-test or Mann–Whitney *U* test for continuous variables. Multivariable linear regression was used to assess differences in HRQoL domains across case groups adjusting for the potential confounding effects of selected variables and multivariable logistic regression was used to estimate the odds in the case of binary variables. Spearman rank correlation was used to investigate diagnostic delay and HRQoL. Potential confounding variables were decided *a priori* (sociodemographic variables) or were those variables that were found to be confounding variables after adjustment (clinical variables).

### Ethics

The study was approved by the Oxford Tropical Research Ethics Committee (OxTREC) of Oxford University (OxTREC reference: 37-17) and the Eastern Mediterranean University Ethics Committee (ETK00-2017-0240).

## Results

### Endometriosis prevalence, demographics and pain symptomatology

The self-reported prevalence of endometriosis was 5.1% [95% confidence interval (CI): 4.7–5.7, *n* = 395/7646] and the prevalence of endometriomas picked up as part of the USS was 2.7% (95% CI: 1.7–4.3, 18/663). Of the 42 self-reported endometriosis cases who also had an USS, 3 (7.1%) had evidence of an endometrioma on their scan. The overall prevalence of endometriosis in this study was 5.4% (95% CI: 4.9–5.9, *n* = 410). There was no significant difference in any demographics between the incident endometrioma (*n* = 15) cases diagnosed at the USS and the self-reported endometriosis (*n* = 395) cases before or after adjustment for age (not shown).

In the endometriosis group, 52.9% (*n* = 217) was characterized as being symptomatic for pain; 90.8% (*n* = 197) dysmenorrhoea, 11.5% (*n* = 25) dyspareunia and 24.4% (*n* = 53) non-cyclic pelvic pain. Of the 7236 controls, 40.5% (*n* = 2927) was characterized as symptomatic pain controls and 59.6% (*n* = 4314) was characterized as asymptomatic pain controls (Supplementary figure S1).

Average age of women at the time of enrolment with endometriosis was 37.8 (SD = 8.3) compared with 32.8 (SD = 8.7) and 39.6 (SD = 9.4) in symptomatic and asymptomatic controls, (*P* < 0.001). After adjustment for age, the proportion of women in paid employment was highest in endometriosis cases (86.8%) and cases were more likely to have a higher education compared with controls (*P* < 0.001) (table 1).

**Table 1:** Demographic characteristics and comparisons of the endometriosis case group and two control groups

	(A) Endometriosis (*n* = 410)	(B) Symptomatic pain controls (*n* = 2927)	Crude *P*-value (A vs. B)	Age adj. *P*-value (A vs. B)	(C) Asymptomatic controls (*n* = 4314)	Crude *P*-value (A vs. C)	Age adj. *P*-value (A vs. C)
Demographics							
Age, mean (SD)	37.8 (8.3)	32.8 (8.7)	<0.001	–	39.6 (9.4)	<0.001	–
Ethnicity, *n* (%)			0.041	0.053		0.27	0.231
Turkish Cypriot	310 (77.7)	2053 (72.2)			3015 (74.5)		
Turkish	69 (17.3)	625 (22.0)			831 (20.5)		
Other/mixed	20 (5.0)	166 (5.8)			202 (5.0)		
Residence type, *n* (%)			0.822	0.745		0.287	0.327
City	200 (48.8)	1408 (48.2)			1986 (46.0%)		
Village	210 (51.2)	1514 (51.8)			2328 (54.0%)		
Residence, *n* (%)			0.083	0.242		0.074	0.047
Famagusta	66 (16.1%)	641 (21.9%)			904 (21.0%)		
Kyrenia	68 (16.6%)	467 (16.0%)			728 (16.9%)		
Lefke	17 (4.1%)	91 (3.1%)			153 (3.5%)		
Morphou	28 (6.8%)	220 (7.5%)			337 (7.8%)		
Nicosia	212 (51.7%)	1256 (43.0%)			1833 (42.5%)		
Trikomo	19 (4.6%)	247 (8.5%)			359 (8.3%)		
Employment, *n* (%)			<0.001	0.001		<0.001	<0.001
Employed	356 (89.2)	2219 (78.0)			3345 (82.4%)		
Unemployed	43 (10.8)	627 (22.0)			713 (17.6%)		
Education, *n* (%)			0.007	<0.001		<0.001	<0.001
Primary/middle	29 (7.5)	283 (10.1%)			533 (13.4%)		
High/post-secondary	118 (30.4)	947 (33.7%)			1492 (37.4%)		
Undergraduate	152 (39.2)	1066 (37.9%)			1365 (34.2%)		
Postgraduate	89 (22.9)	516 (18.3%)			599 (15.0%)		
Civil status			<0.001	0.195		0.009	0.247
Single	77 (19.5%)	1067 (37.6%)			609 (15.1%)		
Divorced/separated	46 (11.6%)	224 (7.9%)			414 (10.2%)		
Married	272 (68.9%)	1550 (54.6%)			3017 (74.7%)		
Migrant status			0.350	0.116		0.542	0.677
Non-migrant	2104 (74.0%)	304 (76.2%)			1020 (25.2%)		
Migrant	739 (26.0)	95 (23.8%)			3028 (74.8%)		
Short Form McGill Questionnaire (SF-MPQ)							
Affective, mean (SD)	9.0 (4.5)	8.5 (4.0)	0.370	0.158	4.8 (1.5)	<0.001	<0.001
Sensory, mean (SD)	24.6 (9.4)	23.6 (8.1)	0.460	0.245	15.0 (4.0)	<0.001	<0.001
Overall, mean (SD)	33.1 (13.8)	31.9 (11.4)	0.550	0.381	19.8 (5.3)	<0.001	<0.001
Pain catastrophizing scale (PCtS)							
Rumination	9.6 (4.7)	9.6 (4.7)	0.784	0.569	8.0 (4.2)	<0.001	<0.001
Magnification	6.0 (2.8)	6.0 (2.8)	0.759	0.443	5.2 (2.5)	<0.001	<0.001
Helplessness	13.0 (6.0)	12.8 (6.0)	0.489	0.163	10.7 (5.2)	<0.001	<0.001
Overall PCtS	27.6 (12.1)	27.5 (12.1)	0.936	0.381	23.2 (10.6)	<0.001	<0.001
Associated pain							
Bladder pain	26 (6.3%)	187 (6.4%)	0.964	0.634	42 (1.0%)	<0.001	<0.001
IBS related to non-cyclic pelvic pain	19 (4.6%)	75 (2.6%)	**0.020**	**0.027**	13 (0.3%)	<0.001	<0.001
IBS related to dysmenorrhoea	19 (4.6%)	157 (5.4%)	0.531	0.634	50 (1.2%)	<0.001	<0.001
Migraine	89 (21.7%)	529 (18.1%)	0.079	0.422	528 (12.2%)	<0.001	<0.001
Reproductive							
Parity, *n* (%)	288 (70.9%)	1522 (52.6%)	<0.001	0.914	3331 (79.1%)	0.002	0.033
Infertility, *n* (%)^a^	64 (15.6)	206 (7.0%)	<0.001	<0.001	315 (7.3%)	<0.001	<0.001
Treatment							
Ever used hormones	197 (48.1)	690 (23.6)	<0.001	<0.001	880 (20.4)	<0.001	<0.001
Pain killers over the counter^b^	22 (41.5%)	203 (46.4%)	0.505	0.546	–	–	–
Pain killers prescribed^b^	18 (34.0%)	72 (16.4%)	0.002	0.005	–	–	–
Hormones, but pain not alleviated^b^	5 (9.4%)	2 (0.5%)	<0.001	^c^	–	–	–
Hormones, pain was somewhat alleviated^b^	5 (9.4%)	6 (1.4%)	0.003	^c^	–	–	–
Endometriosis treated in last surgery	81 (19.8%)	–	–	–	–	–	–
Never had surgery for endometriosis	104 (25.4%)	–	–	–	–	–	–
Endometriosis diagnosis							
Diagnostic method^d^							
Laparoscopy or other surgery	46 (11.6)	–	–	–	–	–	–
Ultrasound/MRI	331 (83.8)	–	–	–	–	–	–
Based on symptoms	60 (15.2)	–	–	–	–	–	–
Other^e^	17 (4.3)	–	–	–	–	–	–
Missing	4 (1.0)	–	–	–	–	–	–
Symptoms prompting healthcare appointment							
Pain	205 (51.9)	–	–	–	–	–	–
Infertility	23 (5.8)	–	–	–	–	–	–
No symptoms	63 (15.9)	–	–	–	–	–	–
Other^f^	81 (20.5)	–	–	–	–	–	–
Missing	80 (20.3)	–	–	–	–	–	–

Notes: IBS: Irritable bowel syndrome classified using the Rome III criteria.

a Infertility defined as not being able to get pregnant for 6 months or more at any one time.

b Only includes women with pelvic pain above 4 on the NRS; *N* = 53 for endometriosis and 438 for symptomatic controls.

c Sample size too small for age adjustment.

d Participants able to select multiple so percentage do not add up to 100%; Denominator (*n* = 395) excludes the 15 cases picked up as part of the COHERE.

e Other: changes in menstruation, surgery, during general check-up.

f Other: changes in menstruation, during general check-up.

Endometriosis cases had higher pain scores than asymptomatic controls for the three measures of the SF-MPQ, which measures qualitative pain (*P* < 0.001), as well as all components of the PCtS, which measures how women perceive their pain (*P* < 0.001) (table 1). However, scores did not differ between endometriosis cases and symptomatic controls. Though the overall PCtS mean score did not reach the clinical cut-off of 30 (which represents a clinically relevant level of catastrophizing), 48.3% of endometriosis cases scored 30 or more, compared with 47.3% of the symptomatic controls.

### Bladder pain, irritable bowel syndrome, migraine and psychological disorders

Endometriosis cases were more likely to experience bladder pain compared with asymptomatic pain controls (6.3% vs. 1.0%, *P* < 0.001) and have a higher prevalence of IBS symptoms related to pelvic pain (4.6%) compared with both symptomatic (2.6%, *P* = 0.027) and asymptomatic (0.3%, *P* < 0.001) controls (table 1).

The odds of endometriosis cases being diagnosed with anxiety (8.5%) were 1.56 (95% CI: 1.03–2.38) higher than the symptomatic controls (5.4%, *P* = 0.034) and 1.95 (95% CI: 1.30–2.92) times higher than the asymptomatic controls (3.8%, *P* = 0.001). For depression, 5.4% (*n* = 22) of endometriosis cases reported depression, 4.1% (*n* = 121) of the symptomatic controls and 2.7% (*n* = 116) of the asymptomatic controls.

Odds of migraine in endometriosis cases (21.7%, *n* = 89) compared with asymptomatic controls (12.2%, *n* = 528) was 1.81 (95% CI: 1.39–2.35, *P* < 0.001) (table 2).

**Table 2:** Odds ratio of anxiety, depression and migraine in women with endometriosis vs. symptomatic controls and asymptomatic controls

	Endometriosis cases vs. symptomatic pain controls				Endometriosis cases vs. asymptomatic controls			
	Odds ratio (95% CI)	*P*-value	Adjusted odds ratio (95% CI)^a^	Adjusted *P*-value^a^	Odds ratio (95% CI)	*P*-value	Adjusted odds ratio (95% CI)^a^	Adjusted *P*-value^a^
Anxiety								
No	1	–	1	–	1	–	1	–
Yes	1.64 (1.12–2.41)	0.011	1.56 (1.03–2.38)	0.034	2.35 (1.61–3.43)	<0.001	1.95 (1.30–2.92)	0.001
Depression								
No	1	–	1	–	1	–	1	–
Yes	1.31 (0.82–2.09)	0.253	0.90 (0.54–1.52)	0.707	2.05 (1.29–3.27)	0.003	1.61 (0.96–2.70)	0.069
Migraines								
No	1	–	1	–	1	–	1	–
Yes	1.25 (0.97–1.62)	0.079	1.05 (0.80–1.37)	0.744	1.99 (1.55–2.56)	<0.001	1.81 (1.39–2.35)	<0.001

a Adjusted for age in years (continuous), ethnicity (categorical), education (categorical), employment (categorical) and civil status (categorical).

### Parity and infertility

There was no significant difference in parity between endometriosis cases and symptomatic controls, but cases were less likely to be parous than asymptomatic controls (70.9% vs. 79.1%, *P* < 0.001) (table 1). Endometriosis cases were significantly more likely to struggle to get pregnant for 6 months or more compared with symptomatic controls (15.6% vs. 7.0%, *P* < 0.001) and asymptomatic controls (7.3%, *P* < 0.001),

### Treatment

Overall hormone use in this cohort of women was low (23.1%, *n* = 1767). Endometriosis cases were significantly more likely to use hormones compared with both symptomatic (48.1% vs. 23.6%, *P* < 0.001) and asymptomatic (20.4%, *P* < 0.001) controls. Endometriosis cases were more likely to use prescribed painkillers compared with symptomatic controls (34.0% vs. 16.4%, *P* = 0.005). Under 20% (19.8%, *n* = 81) of endometriosis cases reported to have had their endometriosis treated during their most recent surgery (table 1).

### Endometriosis diagnosis and diagnostic delay

Majority of the cases were diagnosed via ultrasound/magnetic resonance imaging (83.8%, *n* = 331) (table 1). The most common symptom that led to diagnosis was pelvic pain, with 51.9% (*n* = 205) followed by infertility, 5.8% (*n* = 23). There were no significant differences in demographics between women who reported being diagnosed with endometriosis surgically vs. those who did not report to be diagnosed with endometriosis surgically (data not shown).

The median symptom onset age was 25 years [interquartile range (IQR) = 10] and the median diagnosis age was 26 years (IQR = 10), giving a median diagnostic delay of 1 year. Given the high age at symptom onset, it was hypothesized that women in this population have low awareness of endometriosis symptoms, specifically pain. Examining the pain-related variables confirmed this; for dysmenorrhoea, the earliest median age of pain was 14 years (IQR = 5.5), for non-cyclic pelvic pain, the earliest median age of pain was 21 years (IQR = 13) and for dyspareunia, the earliest median age of pain was 25 years (IQR = 8). Diagnostic delay was recalculated for 76.5% (*n* = 199) of the endometriosis cases giving an overall median diagnostic delay of 7 years (IQR = 15.5) (*n* = 292, which includes the 199 participants who had diagnostic delay recalculated and 93 participants who did not have diagnostic delay recalculated).

### Quality of life

After adjustment for demographics and age of first period pain, the only significant differences in HRQoL between endometriosis cases and symptomatic controls were observed for the role physical subscale (76.83 vs. 80.50 *P* = 0.022) and for the overall PCS measure (46.79 vs. 48.54, *P* = 0.034), suggesting that endometriosis cases had worse physical health compared with symptomatic controls (table 3). Endometriosis cases scored significantly lower on all subscales and overall summary measures compared with asymptomatic controls. Stratification based on experiencing one type of pain or more than one type of pain showed that women experiencing more than one pain had significantly impaired HRQoL for all domains (*P* < 0.001).

**Table 3:** Association between endometriosis and HRQoL compared with symptomatic pain controls and asymptomatic pain controls

	Endometriosis (*n* = 368)	Symptomatic controls (*n* = 2749)	*P*-value			Asymptomatic controls (*n* = 3972)	*P*-value		Experienced one pain only (n = 2563)	Experienced more than one pain (*n* = 576)	*P*-value	
	Mean (SD)	Mean (SD)	Crude	Adjusted^a^	Further adjusted^b^	Mean (SD)	Crude	Adjusted^a^	Mean (SD)	Mean (SD)	Crude	Adjusted^a^
Physical functioning (PF)	87.39 (14.98)	89.05 (15.40)	0.041	0.640	0.899	87.84 (16.20)	0.593	0.022	90.05 (14.71)	83.79 (17.38)	<0.001	<0.001
Role Physical (RP)	76.83 (23.76)	80.50 (22.89)	0.003	0.190	0.022	81.14 (22.62)	<0.001	<0.001	81.79 (22.42)	72.65 (24.18)	<0.001	<0.001
Bodily pain (BP)	63.75 (24.48)	65.42 (24.55)	0.831	0.843	0.274	70.54 (24.0)	<0.001	<0.001	66.63 (24.28)	57.17 (24.27)	<0.001	<0.001
General health (GH)	61.49 (20.14)	61.44 (20.54)	0.962	0.926	0.810	65.62 (19.08)	<0.001	<0.001	62.44 (20.27)	56.02 (21.25)	<0.001	<0.001
Vitality (VT)	56.58 (21.11)	54.47 (20.64)	0.058	0.314	0.315	59.66 (20.46)	0.005	0.001	55.77 (20.56)	48.55 (20.33)	<0.001	<0.001
Social functioning (SF)	74.16 (23.74)	74.67 (23.91)	0.691	0.734	0.368	79.56 (22.55)	<0.001	<0.001	76.23 (23.48)	66.24 (24.53)	<0.001	<0.001
Role emotional (RE)	75.08 (24.09)	73.96 (24.51)	0.390	0.572	0.901	79.44 (23.15)	<0.001	<0.001	75.59 (24.08)	65.69 (25.16)	<0.001	<0.001
Mental health (MH)	62.87 (19.80)	61.09 (19.88)	0.094	0.141	0.250	66.20 (19.49)	0.001	0.001	62.57 (19.43)	54.00 (20.47)	<0.001	<0.001
PCS^c^	46.79 (8.62)	48.54 (8.84)	<0.001	0.178	0.034	48.31 (8.83)	0.002	0.004	48.90 (8.57)	45.86 (9.79)	<0.001	<0.001
MCS^c^	46.45 (10.96)	44.76 (11.15)	0.006	0.071	0.165	48.10 (10.61)	0.005	0.010	45.56 (10.91)	41.16 (11.49)	<0.001	<0.001

Notes: MCS, mental component score; PCS, physical component score; SD, standard deviation.

a Adjusted for age in years (continuous), ethnicity (categorical), education (categorical), employment (categorical), civil status (categorical).

b Additionally adjusted for age of first period pain in years (continuous).

c Calculated using normative values from the Third Oxford Health and Lifestyles survey.

### Work productivity impairment and economic burden

Compared with asymptomatic controls, women with endometriosis had significantly lower presenteeism (*P* < 0.001), overall work productivity loss (*P* < 0.001) and activity impairment (*P* < 0.001). Levels of absenteeism in this sample in general were low (Supplementary table S1).

Women with endometriosis incurred an annual cost of $9864.35 (95% CI: $8811.55–10917.15), which was slightly less than the symptomatic controls who incurred an annual cost of $10429.68 (95% CI: $10004.18–$10855.17) (*P* = 0.332). Asymptomatic controls incurred significantly less costs per year compared with the endometriosis cases (*P* < 0.001) (table 4). As pain severity increased, the total annual cost incurred also increased (Supplementary table S2). For women with endometriosis, total cost per annum was on average highest between the ages of 36 and 45 at $9389.21 (95% CI: $7718.75–$11059.67) and lowest in ages 46 and 55 at $6628.53 (95% CI: $4652.91–$8604.15) (Supplementary table S3). Total annual cost for women aged 46–55 in the symptomatic pain control group was higher than that of the endometriosis group at $10156.58 (95% CI: $8800.24–$11512.92) with costs remaining constant over the life-course for the asymptomatic pain control group (Supplementary table S3).

**Table 4:** Cost of health and productivity measures in endometriosis cases, symptomatic controls and asymptomatic controls overall per annum

All	A) Endometriosis: *n* = 356, mean (95% CI)	B) Symptomatic controls: *n* = 2219, mean (95% CI)	C) Asymptomatic controls: *n* = 3345, mean (95% CI)	*P*-value (A vs. B)	*P*-value (A vs. C)
Health costs					
Primary care	724.67 (525.69–923.64)	402.95 (365.74–440.16)	289.42 (269.92–308.92)	<0.001	<0.001
Secondary care	1519.63 (1295.92–1743.34)	1175.07 (1085.54–1264.59)	955.75 (887.78–1023.73)	0.005	<0.001
Total	2244.30 (1934.06–2554.54)	1578.02 (1475.85–1680.18)	1245.17 (1169.37–1320.97)	<0.001	<0.001
Productivity costs					
Absenteeism	523.32 (364.74–681.90)	572.90 (507.18–638.62)	473.98 (412.04–535.93)	0.581	0.623
Presenteeism	7096.73 (6187.13–8006.34)	8278.76 (7904.09–8653.44)	4868.48 (4618.23–5118.72)	0.021	<0.001
Total	7620.05 (6667.88–8572.23)	8851.66 (8456.30–9247.03)	5342.46 (5074.14–5610.78)	0.023	<0.001
Grand total	9864.35 (8811.550–10917.15)	10429.68 (10004.18–10855.17)	6587.63 (6294.19–6881.08)	0.332	<0.001

Notes: Currency is International Currency ($). CI, confidence interval.

## Discussion

This is the first study that has estimated the prevalence, diagnostic delay, effects on quality of life, symptomatic and economic burden of endometriosis in Northern Cyprus. This research adds to the existing women’s health research focused on understudied populations. Over two-thirds of women reported that their endometriosis symptoms started at the same age that they received their diagnosis and a low percentage of endometriosis cases reported to have ever used hormones. This suggests a lack of awareness of endometriosis and its symptoms in both the public and clinicians, as well as an underutilization of common first-line treatments for this condition.

The self-reported prevalence of endometriosis was 5.4% in reproductive-aged women, extrapolating to approximately 7500 women living in Northern Cyprus. This prevalence is similar to other published studies that have used similar sampling methods.^28^ Although self-reported diagnostic data may not be ideal, research has shown that women self-report endometriosis with reasonable accuracy (>70%). Incidence of endometrioma was 2.7%. Ultrasounds that are performed by sonographers/clinicians who are not experienced are more likely to be falsely negative^29^ so the low incidence in this study may reflect a lack of specialized healthcare providers in Northern Cyprus. A negative imaging result does not rule out endometriosis so this estimate of prevalence of endometriosis and endometriomas in COHERE is likely underestimated. Only a small number of women were reported to have been diagnosed with endometriosis surgically (12%), most likely because specialized endometriosis laparoscopy is limited in Northern Cyprus.

Endometriosis cases were on average older and more educated than women in the symptomatic control group and given the average age of diagnosis was 26 years and there was high prevalence of pain symptoms in the control group, there are likely undiagnosed cases in this group. Based on self-reported symptom onset age and date diagnosed to calculate diagnostic delay, the mean delay of diagnosis was 1.6 years, which is much lower than other published studies.^8^^,^^9^ Recalculating diagnostic delay using the reported age at which pain symptoms first started gave an average delay of 7 years, similar to worldwide estimates. In addition to this use of hormones to treat endometriosis in this study was low, but similar to research in the UAE, Latin America and Spain.^30^ ESHRE guidelines^31^ state that hormone treatment should be offered to women who are experiencing endometriosis-associated pain. Use of pain medications in this study was low in general, and these findings again highlight opportunities for endometriosis awareness campaigns targeted at both clinicians and the public to improve the quality of life.

There was no significant difference in bladder pain prevalence between endometriosis cases and symptomatic pain controls, suggesting bladder pain may be a co-occurrence in people with pelvic pain rather than strictly related to endometriosis, due to heightened pain sensitivities. Studies^32^ that have shown that bladder pain is more common in individuals with endometriosis have failed to have an appropriate control group, which is a strength of our study. Similarly, we saw no significant difference between endometriosis cases and symptomatic pain controls for IBS related to menstrual pain, but we did see that endometriosis cases had a significantly higher prevalence of IBS symptoms relating to non-cyclic pelvic pain, compared with symptomatic controls. Endometriosis and IBS share several features such as low-grade inflammation and visceral hypersensitivity,^33^ with endometriosis often being misdiagnosed as IBS.^34^

Compared with asymptomatic controls, endometriosis cases had a significantly higher frequency of migraine and anxiety, but not depression. Increased co-occurrence of migraine with endometriosis has been demonstrated in previous studies^35^ and it is believed that the central sensitization theory, which occurs when neural signalling within the central nervous system that is responsible for pain hypersensitivity is amplified,^31^ for chronic pelvic pain contributes to this increased risk of migraines. Indeed, we saw no significantly increased risk of migraine for women with endometriosis when compared with our symptomatic pain control group. The prevalence of anxiety in women with endometriosis was 8.5%, lower than other worldwide estimates,^9^ but the prevalence of anxiety in the asymptomatic group (3.9%) was also low. Therefore, it is likely that the prevalence of both anxiety and depression is underreported in this sample of women, potentially due to a perceived stigma in this population around mental health.^36^ Endometriosis cases were more likely to report that they had received an anxiety diagnosis compared with both control groups, suggesting endometriosis-specific factors are responsible, rather than experience of pain.

In line with a number of other studies,^8^^,^^9^ we saw that women with endometriosis had significantly impaired HRQoL for all domains compared with asymptomatic controls. Contrary to other studies, we did not see the same pattern when comparing to the symptomatic control group, which might be because some women in our case group do not currently have ‘active’ endometriosis and are therefore not experiencing symptoms. Like the GSWH,^8^ we saw that our symptomatic pain control group had slightly lower scores for some of the mental health subscales. Women experiencing more than one pelvic pain type had significantly impaired HRQoL scores compared with women experiencing one pain.

Absenteeism from work was generally low in this population, but the presenteeism of 25.8% is in line with previous estimates. Women in the symptomatic control group had a higher productivity loss through presenteeism. We estimate that endometriosis costs Int$9864.35 to both the individual and society, lower than Australia (Int$20 898)^37^ and the USA ($16 573).^38^ Differences in methodology, the fact that Northern Cyprus offered limited endometriosis specialized laparoscopy, which is a costly treatment, not having salary information for participants and uncontrolled inflation of the Turkish Lira make direct comparisons with other studies difficult. However, like other studies, we saw that loss of productivity at work was the biggest component of overall annual cost. For women with endometriosis, cost was lower among women younger than 35 compared with women aged 35 or older, but this was not the case for the symptomatic control group. We hypothesize that this group of undiagnosed women who suffer from pain continue to incur costs after the age of 35, whereas endometriosis cases have received a diagnosis and are able to manage their symptoms better. These results coupled with the low use of hormones in this population reveal a missed opportunity for both potentially relieving the symptoms experienced by women, increasing productivity in the workplace, and enhancing the economy.

Strengths of our study include its large sample size and the fact that it is broadly representative of the most recent Census estimates. Our multiple control groups have allowed us to investigate whether potential associations are driven by endometriosis or by pain symptoms associated with the disease. Our study relies on self-reported data, which is prone to recall bias; however, we minimized this bias for our work productivity and economic burden calculations by asking questions relating to the past 4 weeks. We were unable to verify self-reported data due to the lack of health registries across the region, so it is likely we have undiagnosed cases in our control groups. However, this would bias estimates towards the null rather than create spurious associations. The cross-sectional design of our study means that we cannot infer causality or temporality. However, since there is a lack of data on endometriosis from the Eastern Mediterranean region, our study helps to fill a gap in endometriosis research in non-Western populations.

For the first time, we have estimated the prevalence of endometriosis, its symptoms, diagnostic delay, economic burden and how it impacts the quality of life for women in Northern Cyprus. Our results show that increased awareness in both the public and medical community is needed to lead to earlier diagnoses, reduced health impairment and improved work productivity. In addition to promoting evidence-based reproductive medicine in the Eastern Mediterranean region, the results form the basis for targeted follow-up studies including investigation of population-specific environmental and genetic factors that contribute to disease risk and pelvic pain.

## Supplementary Material

ckad216_Supplementary_Data

## Data Availability

There are ethical restrictions on sharing the data publicly as not all study participants consented to data being made publicly available. A subset of anonymous data can be requested from Dr. Kurtis Garbutt, email address: kurtis.garbutt@wrh.ox.ac.uk.
Key points This is the first study estimating prevalence and characterizing endometriosis patients and those individuals suffering from pelvic pain in an understudied population from an underinvestigated region. Prevalence of endometriosis is estimated at 5.4% with a 7-year diagnostic delay in Northern Cyprus with significantly impaired health-related quality of life and a high symptomatic and economic burden. A substantial proportion of individuals without endometriosis diagnosis also reported moderate/severe pelvic pain. Coupled with low endometriosis prevalence, it is hypothesized that there are many undiagnosed endometriosis cases within this population. Those individuals with moderate/severe pelvic pain have a worse quality of life and their economic burden does not decrease with age compared with endometriosis cases. Existing pain management options such as the use of hormones are severely underutilized in the population—results showcase the need for increased awareness in both the public and the medical community to lead to earlier diagnoses, reduced health impairment and improved work productivity.
