# Supplementary material for: Prevalence, diagnostic delay and economic burden of endometriosis and its impact on quality of life: results from an Eastern Mediterranean population
Source: Eur J Public Health. 2023 Dec 9;34(2):244–52. doi: 10.1093/eurpub/ckad216 (PMC10990517; doi:10.1093/eurpub/ckad216)
Supplement: ckad216_Supplementary_Data [file ckad216_supplementary_data.zip › ckad216_Supplementary_Data/ejph-2023-09-om-0512-File002.pdf]

Supplementary Table 1. Work productivity and activity impairment in women with endometriosis compared to symptomatic and asymptomatic controls.

	Endometriosis, mean (SD) (n=311)	Symptomatic controls, mean (SD) (n=2,025)	Unadjusted p-value	Adjusted p-value *	Asymptomatic control (n=2,829)	Unadjusted p-value	Adjusted p-value*
**Absenteeism**	2.2 (5.5)	2.5 (5.7)	0.496	0.111	1.90 (5.6)	0.269	0.451
**Presenteeism**	25.8 (28.4)	27.6 (28.4)	0.254	0.289	18.6 (24.8)	**<0.001**	**<0.001**
**Overall work productivity loss**	27.5 (28.5)	29.7 (28.8)	0.248	0.376	19.7 (25.0)	**<0.001**	**<0.001**
**Activity impairment**	23.4 (26.0)	15.4 (26.7)	0.205	0.725	15.3 (21.8)	**<0.001**	**<0.001**

**Adjusted for age in years (continuous), ethnicity (categorical), education (categorical), employment (categorical), civil status (categorical)*
