# Supplementary material for: Prevalence, diagnostic delay and economic burden of endometriosis and its impact on quality of life: results from an Eastern Mediterranean population
Source: Eur J Public Health. 2023 Dec 9;34(2):244–52. doi: 10.1093/eurpub/ckad216 (PMC10990517; doi:10.1093/eurpub/ckad216)
Supplement: ckad216_Supplementary_Data [file ckad216_supplementary_data.zip › ckad216_Supplementary_Data/ejph-2023-09-om-0512-File003.pdf]

Supplementary Table 2. Cost of health and productivity measures by pain severity when considering period pain at its worst in the last 12 months. Currency is International Currency ($).

Pain severity		Minimum (0-2) (n=392)	Mild (3-5) (n=1,020)	Moderate (6-8) (n=1,873)	Severe (9-10) (n=1,602)
**Health costs**	Primary care	53.62 (191.18-316.06)	270.87 (237.55-304.18)	382.99 (355.69-410.29)	489.07 (424.59-553.55)
	Secondary care	723.76 (569.07-878.44)	858.18 (778.18-938.18)	1,095.98 (997.48-1,194.47)	1,401.96 (1,280.96-1,522.97)
	Total	977.38 (805.24-1,149.51)	1,129.05 (1,036.56-1,221.53)	1,478.97 (1,369.20-1,588.73)	1,891.03 (1,746.82-2,035.24)
**Productivity costs**	Absenteeism	509.60 (299.18-720.02)	500.54 (390.48-610.59)	544.0 (465.91-622.08)	542.30 (466.18-618.41)
	Presenteeism	3,678.16 (3,063.63-4,292.69)	5,143.12 (4,712.33-5,573.90)	6,682.50 (6,318.21-7,046.79)	8,367.41 (7,903.95-8,830.88)
	Total	4,187.76 (3,516.28-4,859.24)	5,643.65 (5,177.94-6,109.37)	7,226.50 (6,839.94-7,613.05)	8,909.71 (8,423.44-9,395.98)
	**Grand total**	5,165.14 (4,449.56-5,880.71)	6,772.70 (6,290.26-7,255.15)	8,705.46 (8,284.29-9,126.63)	10,800.74 (10,270.00-11,331.49)
