# Supplementary material for: Prevalence, diagnostic delay and economic burden of endometriosis and its impact on quality of life: results from an Eastern Mediterranean population
Source: Eur J Public Health. 2023 Dec 9;34(2):244–52. doi: 10.1093/eurpub/ckad216 (PMC10990517; doi:10.1093/eurpub/ckad216)
Supplement: ckad216_Supplementary_Data [file ckad216_supplementary_data.zip › ckad216_Supplementary_Data/ejph-2023-09-om-0512-File004.pdf]

Supplementary Table 3. Cost of health and productivity measures in endometriosis cases, symptomatic controls and asymptomatic controls overall per annum, by age group. Currency is International Currency ($).

		Endometriosis: n=356 Mean (95% CI)	Symptomatic controls: n=2,219 Mean (95% CI)	Asymptomatic controls: n=3,345 Mean (95% CI)
**Age 18-25**		**Endometriosis, n=18**	**Symptomatic controls, n=351**	**Asymptomatic controls, n=167**
**Health costs**	Primary care	420.10 (172.99-667.21)	276.70 (218.21-335.19)	206.90 (137.86-275.94)
	Secondary care	1,711.91 (883.25-2,540.58)	1,006.29 (848.94-1,163.64)	950.12 (655.57-1,244.68)
	Total	2,132.01 (1,161.69-3,102.33)	1,282.99 (1,108.30-1,457.68)	1,157.03 (843.71-1,470.34)
**Productivity costs**	Absenteeism	206.84 (65.63-479.32)	412.63 (301.84-523.41)	756.73 (378.99-1,134.46)
	Presenteeism	8,403.58 (5,161.62-11,645.53)	7,858.01 (6,929.91-8,786.11)	5,222.66 (4,071.10-6,374.22)
	Total	8,610.42 (5,403.72-11,817.11)	8,270.63 (7,308.12-9,233.15)	5,979.39 (4,734.68-7,224.09)
	**Grand total**	10,742.43 (7,258.69-14,226.18)	9,553.62 (8,553.59-10,553.66)	7,136.41 (5,817.47-8,455.35)
**Age 26-35**		**Endometriosis, n=125**	**Symptomatic controls, n=958**	**Asymptomatic controls, n=951**
**Health costs**	Primary care	1,000.49 (603.65-1,397.33)	408.02 (369.81-446.23)	354.72 (316.23-393.20)
	Secondary care	1,727.63 (1,325.46-2,129.79)	1,183.89 (1,051.11-1,316.68)	1,006.37 (877.93-1,134.80)
	Total	2,728.11 (2,161.26-3,294.97)	1,591.92 (1,445.49-1,738.34)	1,361.08 (1,217.69-1,504.47)
**Productivity costs**	Absenteeism	703.39 (369.54-1,037.25)	475.10 (397.73-552.46)	396.89 (308.73-485.05)
	Presenteeism	8,728.28 (7,122.48-10,334.07)	8,479.35 (7,912.78-9,045.92)	4,974.80 (4,515.34-5,434.26)
	Total	9,431.67 (7,722.80-11,140.54)	8,954.45 (8,363.76-9,545.14)	5,371.69 (4,888.16-5,855.23)
	**Grand total**	12,159.78 (10,261.25-14,058.32)	10,546.36 (9,911.18-11,181.55)	6,732.77 (6,203.50-7,262.05)
**Age 36-45**		**Endometriosis, n=140**	**Symptomatic controls, n=687**	**Asymptomatic controls, n=1,234**
**Health costs**	Primary care	694.73 (2,161.26-1,045.59)	476.05 (375.11-576.98)	283.35 (250.55-316.14)
	Secondary care	1,431.08 (1,039.82-1,822.35)	1,235.57 (1,043.29-1,427.84)	902.91 (786.40-1,019.41)
	Total	2,125.82 (1,573.41-2,678.22)	1,711.61 (1,483.86-1,939.37)	1,186.25 (1,053.62-1,318.88)
**Productivity costs**	Absenteeism	504.78 (264.36-745.19)	720.02 (565.72-874.32)	427.45 (324.86-530.05)
	Presenteeism	6,758.62 (5,262.96-8,254.28)	8,371.56 (7,690.08-9,053.05)	4,532.87 (4,136.61-4,929.14)
	Total	7,263.40 (5,735.37-8,791.42)	9,091.58 (8,359.04-9,824.12)	4,960.32 (4,530.44-5,390.20)
	**Grand total**	9,389.21 (7,718.75-11,059.67)	10,803.20 (9,997.81-11,608.59)	6,146.57 (5,668.85-6,624.30)
**Age 46-55**		**Endometriosis, n=73**	**Symptomatic controls, n=223**	**Asymptomatic controls, n=993**
**Health costs**	Primary care	384.87 (267.74-502.01)	354.69 (288.43-420.95)	248.30 (214.60-282.00)
	Secondary care	1,285.89 (952.15-1,619.63)	1,216.38 (980.14-1,452.63)	973.91 (855.99-1,091.83)
	Total	1,670.76 (1,294.88-2,046.65)	1,571.08 (1,310.71-1,831.44)	1,222.21 (1,094.25-1,350.16)
**Productivity costs**	Absenteeism	328.57 (102.20-554.95)	792.09 (551.09-1,033.09)	558.09 (431.36-684.81)
	Presenteeism	4,629.19 (2,959.00-6,299.39)	7,793.41 (6,593.18-8,993.65)	5,124.15 (4,637.62-5,610.67)
	Total	4,957.77 (3,158.80-6,756.73)	8,585.50 (7,298.29-9,872.72)	5,682.23 (5,160.09-6,204.38)
	**Grand total**	6,628.53 (4,652.91-8,604.15)	10,156.58 (8,800.24-11,512.92)	6,904.44 (6,340.69-7,468.18)
