# Supplementary material for: Prevalence, diagnostic delay and economic burden of endometriosis and its impact on quality of life: results from an Eastern Mediterranean population
Source: Eur J Public Health. 2023 Dec 9;34(2):244–52. doi: 10.1093/eurpub/ckad216 (PMC10990517; doi:10.1093/eurpub/ckad216)

Figure 1. Flow-chart depicting ascertainment of case and control groups. Symptomatic cases and controls were defined as those who scored above 4 on the numerical rating scale for at least one of the following: dysmenorrhea, dyspareunia, pelvic pain

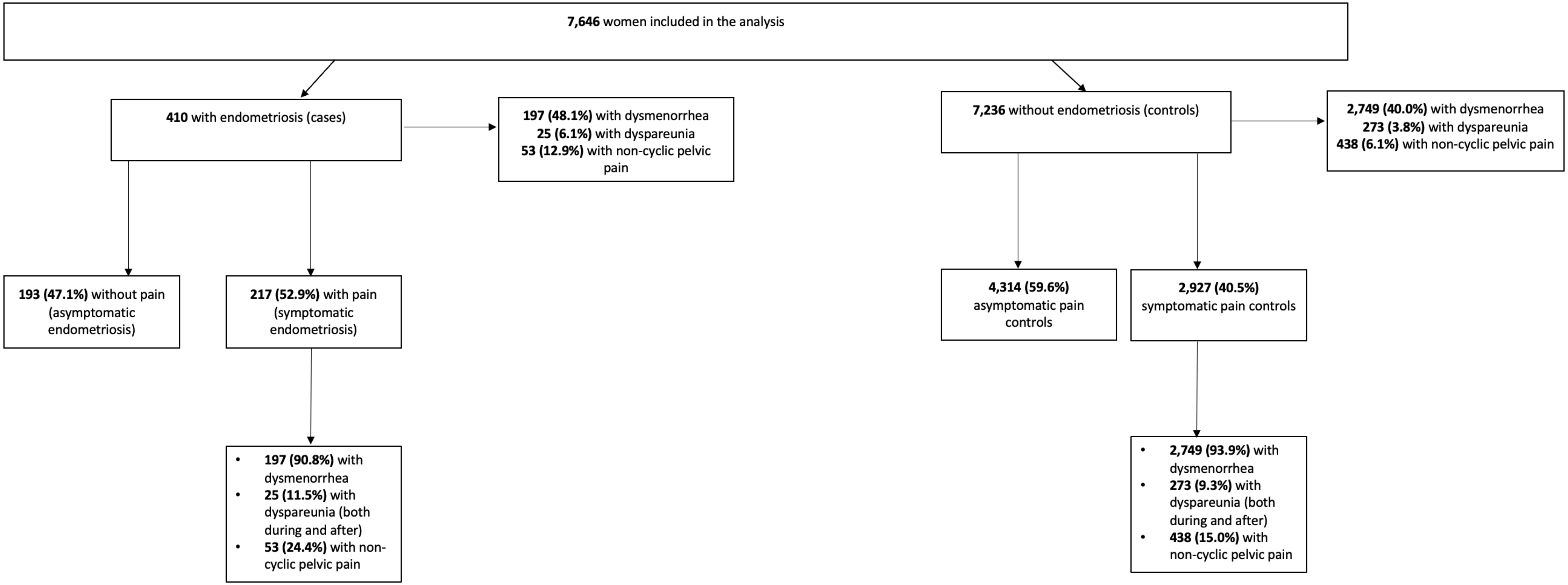

Supplement: ckad216_Supplementary_Data [file ckad216_supplementary_data.zip › ckad216_Supplementary_Data/ejph-2023-09-om-0512-File005.pdf]
