# Supplementary material for: Prevalence, diagnostic delay and economic burden of endometriosis and its impact on quality of life: results from an Eastern Mediterranean population
Source: Eur J Public Health. 2023 Dec 9;34(2):244–52. doi: 10.1093/eurpub/ckad216 (PMC10990517; doi:10.1093/eurpub/ckad216)
Supplement: ckad216_Supplementary_Data [file ckad216_supplementary_data.zip › ckad216_Supplementary_Data/ejph-2023-09-om-0512-File006.pdf]

Supplementary information

Assessment of work productivity and activity impairment

The Work Productivity and Activity Impairment General Health (WPAI:GH) questionnaire was incorporated into the baseline questionnaire and was used to assess the impact of symptoms on absence from work (absenteeism) and reduced productivity whilst working (presenteeism) in endometriosis cases, symptomatic and asymptomatic controls. Questions were asked with regards to the past 4 weeks since endometriosis symptoms can fluctuate across the menstrual cycle. Analysis of the WPAI:GH followed standard methods for the calculation of the dimensions[1]. Absenteeism was calculated as [hours missed due to symptoms/(hours missed due to symptoms + hours actually worked)] x 100. Presenteeism was calculated as [reduced productivity while working] x 100. Overall productivity loss was calculated as [((hours missed due to symptoms + (percent reduced productivity while working x hours actually worked))/ (hours missed due to symptoms + hours actually worked)] x 100. Mean percentage values for these dimensions were compared for working women (employed/self- employed) between endometriosis cases, symptomatic and asymptomatic control groups.

Economic burden

Costs included both direct health care costs[2] and indirect costs related to loss of productivity or time off work. Costs relating to care were not calculated given the small number of individuals who reported to have a carer to assist them with their daily activities (n=63/7,646; 0.82%). Since this was a prevalence analysis, costs were estimated regardless of the time of diagnosis. Average per-person costs were estimated separately for (i) endometriosis cases, (ii) symptomatic pain controls and

(iii) asymptomatic pain controls. Costs were estimated in Turkish Lira using 2022 prices and then converted into International Dollars (Int $), which are tied to the US dollar by dividing the price in Turkish Lira by 2.61, which was the conversion factor taken from the World Bank at the time of analysis[3].

Costs were extrapolated to estimated costs over one year; the technique used was specific to the type of cost considered, outlined below:

Direct costs

<u>Primary care</u>

Costs included in primary care were pharmaceuticals and specialist visits. For medication relating to period pain, information on whether the pain medication was bought over the counter or obtained via prescription was gathered and an average dosage was assumed. For medication relating to pelvic pain, additional information on how long participants had this medication for in the last 3 months was collected and calculated to give a total time per 3 months, as follows – (i) less than one day ~ 0.5, (ii) one day – 1, (iii) two to three days ~ 2.5, (iv) one day a week ~ 4, (v) more than one day a week ~ 3.5 and (vi) every day ~ 91. For hormones. If participants were asked if they had ever used selected hormones and if a respondent answered yes to either the combined birth control pill or the progesterone only pill, it was assumed that this was for at least one year and the cost was taken into account accordingly.

To extrapolate to annual costs, it was assumed that costs incurred would repeat over the course of the year. Therefore, for period pain, it was assumed women would

have 12 periods in one year, so the cost was multiplied by 12. For pelvic pain, costs were multiplied by 4. Information was collected on specialist visits during the last 6 months, it was assumed these would repeat throughout the year, so this number was multiplied by 2.

<u>Secondary care costs</u>

This includes all hospital in-patient and out-patient care, including diagnostics, surgeries, visits to emergency care and hospitalisations. To extrapolate to annual costs, different assumptions were used dependent on the type of cost incurred. For diagnostics (blood tests, ultrasounds, and MRI/CT scans), use of these was reported in the last 6 months, and it was assumed that this would not be repeated continuously throughout the year, so these estimates were not extrapolated further, similar to the methods by Armour *et al.,* 2019[4].

For surgery costs, the baseline questionnaire collected data on the type and number of surgeries each respondent reported to have had so far in their life. To calculate an annual cost for this, it was assumed that the earliest age any surgery would have taken place was 16 years. For each type of surgery, the following calculation was used:

$$\left(\frac{Number\ of\ surgeries}{Age\ at\ time\ of\ recruitment - 16}\right) x\ price\ of\ surgery$$

For hysterectomies, oophorectomies and tubal ligations, the number of surgeries was replaced with 1 since these surgeries are not able to be repeated. For laparoscopy, it was assumed to be diagnostic for those without endometriosis and

assumed to be an endometrioma excision for those who reported to have endometriosis.

Unit costs for pharmaceuticals and specialist visits were obtained through local knowledge. Costs for surgeries and procedures were obtained from the tariff given by the Turkish Cypriot Medical Association[5].

For fertility treatments, the baseline questionnaire collected data on the type and number of fertility treatments (fertility drugs, IVF, intrauterine insemination (IUI)) each respondent reported to have had so far in their life. To calculate an annual cost, the same calculation was performed as for surgery costs, but rather than using 16 for the earliest age, the earliest age of each fertility treatment in the sample was used i.e., 24 for fertility drugs, 30 for IUI and 25 for IVF.

Indirect costs: productivity impact

Initial impact

A 'human capital approach' was used where lost working time was valued by lost income to each person. Income information was not collected as part of the baseline questionnaire, so the minimum national income of 84,000 Turkish Lira was used, giving a pro-rata daily income of 230 Turkish Lira. Costs were divided into 'Absenteeism' and 'Presenteeism', as detailed previously. Absenteeism is measured by days absent from work which was then multiplied by the pro rate daily income rate and the latter was measured from '*During the four weeks, how much did your symptoms affect your productivity while you were working?*' – responses from 0-10 were converted into associated percentages (e.g. 2 = 20%) and then multiplied by

the daily income. To extrapolate these costs to annual costs, the one-month absenteeism and presenteeism costs were multiplied by twelve.

Outcomes: average costs per patient

The average cost per person was estimated across the categories described above and collated into both an average for the sample as a whole and by age categories (18-25, 26-35, 36-45 and 46-55) as well as by pain severity during the menstrual cycle when the pain was at its worst (Minimal' (1–2), 'Mild' (3–5), 'Moderate' (6–8), 'Severe' (9–10)). Analysis was conducted by endometriosis cases, symptomatic controls, and asymptomatic controls separately. Means and 95% confidence intervals are reported.

Exclusion criteria

To not bias estimates, analysis was restricted to participants who reported to be employed as questions on productivity assume the individual was employed.

References

1. Reilly MC, Zbrozek AS, Dukes EM. The Validity and Reproducibility of a Work Productivity and Activity Impairment Instrument. Pharmacoeconomics. 1993;4: 353–365. doi:10.2165/00019053-199304050-00006
2. Simoens S, Hummelshoj L, D'Hooghe T. Endometriosis: Cost estimates and methodological perspective. Human Reproduction Update. 2007. pp. 395–404. doi:10.1093/humupd/dmm010
3. The World Bank. PPP conversion factor, GDP (LCU per international $) | Data | Table. In: The World Bank [Internet]. 2012 [cited 10 Oct 2022]. Available: http://data.worldbank.org/indicator/PA.NUS.PPP
4. Armour M, Lawson K, Wood A, Smith CA, Abbott J. The cost of illness and economic burden of endometriosis and chronic pelvic pain in Australia: A national online survey. PLoS One. 2019;14. doi:10.1371/journal.pone.0223316
5. Cyprus Turkish Medical Association. Obstetrics and Gynecology - Cyprus Turkish Medical Association. 2022 [cited 10 Oct 2022]. Available: https://www.kttb.org/tarifeler/kadin-hastaliklari/
